# Notes and Comments

**Published:** 1899-06

**Authors:** 


### Notes and Comments.[1]

ARSENIC IN DEVITALIZING PULPS.—The severe pains accompanying applications of arsenic to the dental pulp may be considerably lessened if an equal amount of antipyrin is used in connection with the arsenical paste. The antipyrin reduces the blood-supply, and hence prevents the congestion that invariably results from the use of arsenic.—*Dental Digest.*

---

TINCTURE OF IODINE FOR THE REMOVAL OF DEPOSITS.—Dr. Register says, "Apply the dilute tincture freely to teeth and gums, which will constringe puffy gums, drawing them away from about the teeth and outline deposits, which might otherwise escape detection. It loosens deposits upon the teeth, insinuating itself into minute crevices and rough areas of the crowns. Follow the iodine with ammonia, which forms a colorless solution, leaving the teeth much lighter in color. Clean and polish the surfaces."

---

CATAPHORESIS—LEAKAGE.—Avoid the use of ligatures in applying the rubber-dam, as, unless they are steeped in melted wax or thoroughly saturated with chloro-percha, they become the means of conveying the current to some point where there is gum contact, thus causing leakage. Oxyphosphate, mixed to a creamy consistency and poured around the margins and upon adjoining surfaces, forms a box cavity which is thoroughly insulated.—W. V. B. AMES, *Dental Review.*

---

BLEEDING GUMS.—To obviate bleeding of the gums in crown- and bridge-work, Dr. Conrad E. Wittlaufer, in the *Dental Practi-*

---

[1] The assistant editor solicits contributions for this department,—new methods, new remedies and formulas, or any short practical note which may prove of value to the practitioner or student. Address 1338 Walnut Street, Philadelphia.

*tioner,* says, apply a twenty-five-per-cent. solution of pyrozone. This acts as a styptic, one or two applications rendering the gum perfectly dry for from ten to fifteen minutes.

